# Supplementary material for: Assessing emergency healthcare accessibility in the Salton Sea region of Imperial County, California
Source: PLoS One. 2021 Jun 30;16(6):e0253301. doi: 10.1371/journal.pone.0253301 (PMC8244845; doi:10.1371/journal.pone.0253301)
Supplement: Attachment — Submitted filename: Response to Reviewers.docx [file pone.0253301.s001.docx]

***Response to the editor’s and reviewer’s comments***

*Editorial Board*

PLOS ONE Journal

May 28, 2021

Dear PLOS ONE Editorial Board,

Thank you for inviting me to submit a revised draft of my manuscript entitled, “Assessing emergency healthcare accessibility in the Salton Sea region of Imperial County, California” to *PLOS ONE*.

I am appreciative of the time and effort you and each of the reviewers have dedicated to providing insightful feedback on ways to strengthen my paper. It is with great pleasure that I resubmit my article for further consideration. I have incorporated changes that reflect the detailed suggestions you have provided to the best of my abilities. I have no conflicts of interest to disclose; please address all correspondence concerning this revised manuscript to me at pjutu001@ucr.edu.

I hope that the edits and the responses provided below satisfactorily address all the issues and concerns you and the reviewers have noted. Please find attached the revised manuscript. Again, thank you for giving me the opportunity to strengthen my manuscript with your valuable comments and queries. I have worked diligently to incorporate your feedback and hope that these revisions persuade you to accept my submission.

To facilitate your review of my revisions, the following is a point-by-point response to the questions and comments delivered in your letter dated April 8, 2021.

Sincerely,

Preeti Juturu

B.A. Candidate, Public Policy & Economics

University of California, Riverside Center for Geospatial Sciences

**Journal Requirements:**

**Comment-1.** Please ensure that your manuscript meets PLOS ONE's style requirements, including those for file naming. The PLOS ONE style templates can be found at https://journals.plos.org/plosone/s/file?id=wjVg/PLOSOne_formatting_sample_main_body.pdf and https://journals.plos.org/plosone/s/file?id=ba62/PLOSOne_formatting_sample_title_authors_affiliations.pdf

***Response:*** *Thank you for your feedback. This manuscript has been revised according to the journal style requirements (please see the revised manuscript).*

**Comment-2.** We note that Figures 1, 2, 3 and 5 in your submission contain map images which may be copyrighted. All PLOS content is published under the Creative Commons Attribution License (CC BY 4.0), which means that the manuscript, images, and Supporting Information files will be freely available online, and any third party is permitted to access, download, copy, distribute, and use these materials in any way, even commercially, with proper attribution. For these reasons, we cannot publish previously copyrighted maps or satellite images created using proprietary data, such as Google software (Google Maps, Street View, and Earth). For more information, see our copyright guidelines: http://journals.plos.org/plosone/s/licenses-and-copyright.

We require you to either (a) present written permission from the copyright holder to publish these figures specifically under the CC BY 4.0 license, or (b) remove the figures from your submission:

a. You may seek permission from the original copyright holder of Figures 1, 2, 3 and 5 to publish the content specifically under the CC BY 4.0 license.

We recommend that you contact the original copyright holder with the Content Permission Form (http://journals.plos.org/plosone/s/file?id=7c09/content-permission-form.pdf) and the following text:

“I request permission for the open-access journal PLOS ONE to publish XXX under the Creative Commons Attribution License (CCAL) CC BY 4.0 (http://creativecommons.org/licenses/by/4.0/). Please be aware that this license allows unrestricted use and distribution, even commercially, by third parties. Please reply and provide explicit written permission to publish XXX under a CC BY license and complete the attached form.”

Please upload the completed Content Permission Form or other proof of granted permissions as an "Other" file with your submission.

In the figure caption of the copyrighted figure, please include the following text: “Reprinted from [ref] under a CC BY license, with permission from [name of publisher], original copyright [original copyright year].”

b. If you are unable to obtain permission from the original copyright holder to publish these figures under the CC BY 4.0 license or if the copyright holder’s requirements are incompatible with the CC BY 4.0 license, please either i) remove the figure or ii) supply a replacement figure that complies with the CC BY 4.0 license. Please check copyright information on all replacement figures and update the figure caption with source information. If applicable, please specify in the figure caption text when a figure is similar but not identical to the original image and is therefore for illustrative purposes only.

The following resources for replacing copyrighted map figures may be helpful:

USGS National Map Viewer (public domain):

http://viewer.nationalmap.gov/viewer/

The Gateway to Astronaut Photography of Earth (public domain):

http://eol.jsc.nasa.gov/sseop/clickmap/

Maps at the CIA (public domain):

https://www.cia.gov/library/publications/the-world-factbook/index.html and

https://www.cia.gov/library/publications/cia-maps-publications/index.html

NASA Earth Observatory (public domain): http://earthobservatory.nasa.gov/

Landsat: http://landsat.visibleearth.nasa.gov/

USGS EROS (Earth Resources Observatory and Science (EROS) Center) (public domain): http://eros.usgs.gov/#

Natural Earth (public domain):

http://www.naturalearthdata.com/

***Response:*** *Thank you for bringing this to my attention. The figures have been revised using GQIS, so figures are copyright-free and are formatted as per journal requirements (please see the revised figures).*

**Comment-3.** Please amend either the title on the online submission form (via Edit Submission) or the title in the manuscript so that they are identical.

***Response:*** *Thank you for your feedback. I have incorporated your comments by renaming the manuscript title (please see the revised manuscript).*

**Response to Reviewer #1 comments:**

The paper used RAAM to assess the level of emergency healthcare access in the Salton Sea region of Imperial County. In addition to travel times, the supply and demand for hospitals were taken into account. The method is novel and the study has practical application value. But there are still some problems:

**Comment-1:** Introduction. It should include reviews of other studies assessing the level of emergency healthcare access and explain the gaps to reflect the research value and innovation of this paper.

***Response:*** *Thank you for your valuable insight. I’ve attempted to address these items by discussing existing literature on rural emergency healthcare access. I’ve also included current gaps in existing research and ways in which my work supplements and expands upon current literature. However, I was unable to include many reviews as most work does not focus on rural regions. Therefore, this section in the introduction may be a bit shorter than other works.*

**Comment-2:** In Fig.5, it is suggested to increase the number of grades of the RAAM Scores to make the visualization effect better. The current results seem to be relatively general and cannot correspond well with the results in Fig.3. Also, the clarity of all figures should be improved.

***Response:*** *Thank you for your suggestions. I agree with you and have incorporated this suggestion by re-visualizing my data for improved figure clarity. I have also increased the number of grades of the RAAM values, so they are clearer to visualize and interpret (please see the revised figures).*

**Comment-3:** The paper is of practical value, and the author gives policy suggestion. However, the content and analysis are not rich enough to meet the requirements of a research article. It is recommended to add deeper analysis and interpretation corresponding with the Policy Challenges section. In addition, the innovation of the study needs to be explained: whether the data/indicators are more perfect, or the method is better, etc. Most importantly, what are the supplements to the previous studies in this field. Please elaborate the innovation in the Introduction or Conclusion section.

***Response:*** *Thank you for providing these insights. I have incorporated your comment about policy analysis and interpretation in my work. This has been done by drawing from existing research that assesses emergency healthcare access in rural areas and gauging the effectiveness of the previous healthcare access policies. However, there are certain limitations in doing so as academic literature and research on policy recommendations to address spatial access to rural emergency healthcare facilities are limited in nature. This study arguably seeks to bring attention to the spatial inaccessibility to healthcare facilities in the region at hand and brings awareness to potential solutions that may be deployed to address spatial healthcare accessibility. Therefore, I speak primarily about policy examples that may be helpful in addressing emergency healthcare access. Regarding the study innovation, I attempt to discuss the ways in which my work expands upon current work, and I incorporate this within my materials and methods section in addition to the results and discussion section.*

**Response to Reviewer #2 comments:**

The authors assessed the emergency healthcare accessibility in the Salton Sea region of Imperial County. The research methodologies are reasonable, and the findings are interesting. However, there are still a few aspects that should be improved to make the paper publishable. As the article is coherent and well-organized, I focus here only on some points, which are hopefully easy for the authors to take into account in the revision.

**Comment-1:** North arrow should be added to figures.

***Response:*** *Thank you for your suggestion. I have incorporated this feedback into my newly produced figures (please see the revised figures).*

**Comment-2:** This work used 3:00 to 4:00 PM as a reference time to assess the accessibility. What about accessibility in the normal time? The comparison should be discussed because emergencies are random.

***Response:*** *Thank you for raising this important question. In this work, I use the 3:00 to 4:00 PM reference time as a case study to generate and analyze travel times. Though this is a static time frame, which may arguably be limiting in nature considering the unpredictability of emergency health situations, rural regions such as Imperial County experience constant traffic conditions throughout the year, with routine traffic congestion being seasonal (Gastelle et al., 2017). This would be especially expected out of Imperial County, as it has only experienced a population increase of 3.8% from 2010 to 2019 (U.S. Census Bureau, 2019). The lack of significant population growth, paired with rural road conditions such as lower average saturation flows, indicates that traffic conditions may be constant across days and times with minimal variability. However, to ensure results are generalizable, the RAAM model uses average driving travel times to calculate RAAM values. Therefore, RAAM values are representative of annual and daily fluctuations and can be used to compare case-study results. Interestingly, the travel times choropleth map is closely aligned with the RAAM values map, which may suggest that traffic conditions in the region remain constant. Though that is not necessarily the scope of the study, this correlation has emerged and may be helpful in studying further to determine appropriate policy solutions. I have added this information and its corresponding academic sources to my manuscript to address your question, which is deeply appreciated.*

**Comment-3:** There are also some methods to evaluate accessibility pattern, and the advantages and disadvantages need to be emphasized. Suggest reading, for instance, (1) The influence of high-speed rail on ice–snow tourism in northeastern China. Tourism Management,2020, doi:10.1016/j.tourman.2019.104070. (2) Study on the Impact of High-speed Railway Opening on China's Accessibility Pattern and Spatial Equality[J]. Sustainability, 2018,10,2943. doi:10.3390/su10082943

***Response:*** *Thank you for your feedback and for providing me with these resources. I have had the opportunity to review and draw information from them regarding social demand; this information has been incorporated into the results and discussion section. However, incorporating materials on accessibility patterns and the various methods in-depth may be beyond the scope of this paper. The focus for this work is to assess spatial emergency healthcare access within Imperial County with a particular focus on the Salton Sea region. The emphasis for this study is on the RAAM model and economic demand, as opposed to personal resources or demographics. However, I will be sure to consider it for future work, such as conducting a multivariate analysis of emergency healthcare access patterns and spatial equity.*
